# Supplementary material for: Splice-Junction-Based Mapping of Alternative Isoforms in the Human Proteome
Source: Cell Rep. Author manuscript; Available in PMC 2020 Jan 15. (PMC6961840; doi:10.1016/j.celrep.2019.11.026)
Supplement: 2 [file NIHMS1546469-supplement-2.zip › DF1/PXD006675/AtrialSeptum/AtrialSeptum_27_TPM3_AISEELDHALNDMTSGSQMLKLDK.pdf]

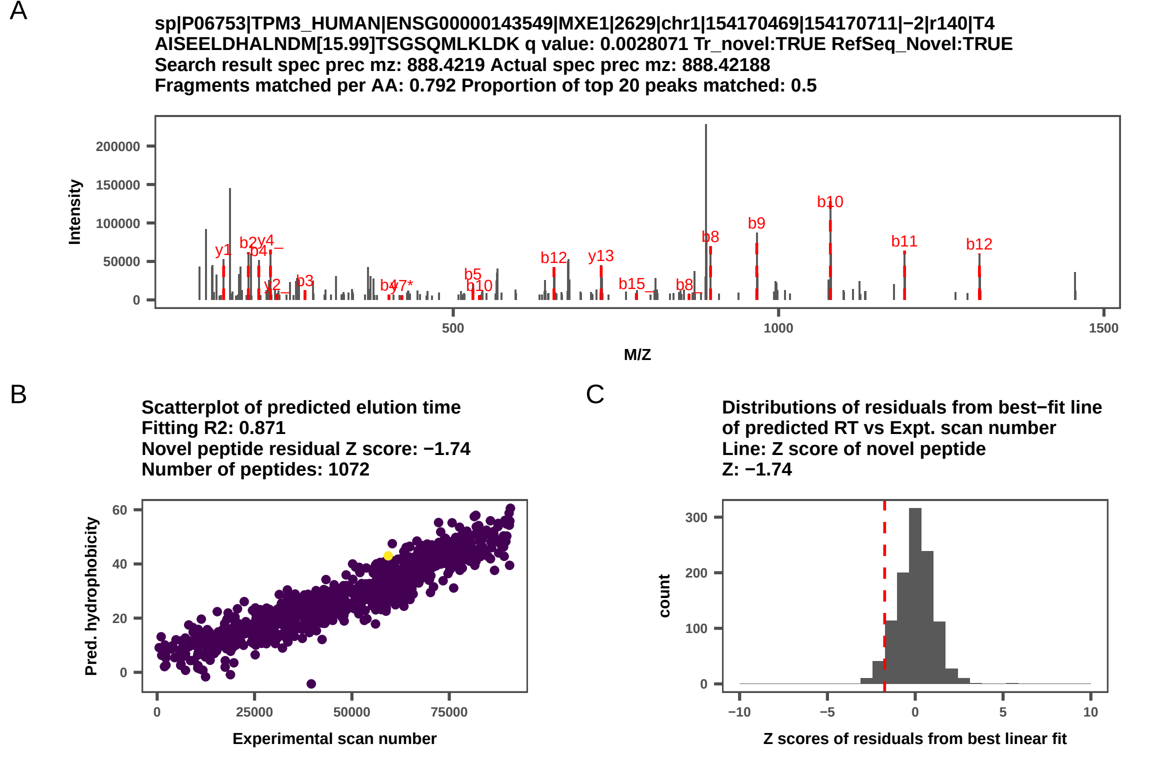
A
sp|P06753|TPM3_HUMAN|ENSG00000143549|MXE1|2629|chr1|154170469|154170711|−2|r140|T4
AISEELDHALNDM[15.99]TSGSQMLKLDK q value: 0.0028071 Tr_novel:TRUE RefSeq_Novel:TRUE
Search result spec prec mz: 888.4219 Actual spec prec mz: 888.42188
Fragments matched per AA: 0.792 Proportion of top 20 peaks matched: 0.5
Intensity
200000
150000
100000
50000
0
y1
b2
y4_
b4_
y2_
b3
b4
y7*
b5
b10
b12
y13
b15_
b8_
b8
b9
b10
b11
b12
500
1000
1500
M/Z
B
Scatterplot of predicted elution time
Fitting R2: 0.871
Novel peptide residual Z score: −1.74
Number of peptides: 1072
Pred. hydrophobicity
60
40
20
0
0
25000
50000
75000
Experimental scan number
C
Distributions of residuals from best−fit line
of predicted RT vs Expt. scan number
Line: Z score of novel peptide
Z: −1.74
count
300
200
100
0
−10
−5
0
5
10
Z scores of residuals from best linear fit
